# Fabrication of g-C_3_N_4_ Nanosheets Anchored With Controllable CdS Nanoparticles for Enhanced Visible-Light Photocatalytic Performance

**DOI:** 10.3389/fchem.2021.746031

**Published:** 2021-10-14

**Authors:** Minggui Wang, Min Wang, Fang Peng, Xiaohuan Sun, Jie Han

**Affiliations:** ^1^ Guangling College, Yangzhou University, Yangzhou, China; ^2^ School of Chemistry and Chemical Engineering, Yangzhou University, Yangzhou, China

**Keywords:** G-C3N4, CdS, hybrids, photocatalyst, visible light

## Abstract

Herein, g-C_3_N_4_/CdS hybrids with controllable CdS nanoparticles anchoring on g-C_3_N_4_ nanosheets were constructed. The effects of CdS nanoparticles on photocatalytic H_2_ production and organic molecule degradation for g-C_3_N_4_/CdS hybrids were investigated. The maximum rate of H_2_ production for g-C_3_N_4_/CdS sample was 1,070.9 μmol g^−1^ h^−1^, which was about four times higher than that of the individual g-C_3_N_4_ nanosheet sample. The enhanced photocatalytic performance for prepared hybrids could be mainly attributed to the following causes: the formed heterojunctions can contribute to the light absorption and separation of photogenerated electrons and holes, the two-dimensional layered structure facilitates the transmission and transfer of electrons, and high specific surface area could provide more exposed active sites.

## Introduction

Semiconductor materials have received widespread attention as promising photocatalysts for clean energy production and environmental problems. (Tong et al., 2012; Chang et al., 2016; Yu et al., 2017; Qi et al., 2018; Cao et al., 2019; Ng et al., 2021). Recently, g-C_3_N_4_ has received considerable attention as a promising photocatalyst, owing to its ease of preparation, high stability, low cost, clean and low toxicity, narrowed bandgap (∼2.7 eV), and special two-dimensional (2D) layered structure (Shi et al., 2016; Teng et al., 2017; Han et al., 2018; Deng et al., 2019; Ong et al., 2020; Vu et al., 2020; Yu et al., 2021); however, diverse drawbacks include poor efficiency of light utilization and low separation of photogenerated charges during the application, making g-C_3_N_4_ less attractive for photocatalyst construction. (Tahir et al., 2014; Liu et al., 2017; Al Marzouqi et al., 2019; Fu et al., 2019; Fan et al., 2020). In addition, bulk g-C_3_N_4_ with a low surface area and irregular morphology prepared through the conventional method leads to the low transfer rate of interfacial charge and poor photocatalytic activity (Teng et al., 2017; Niu et al., 2018).

For the sake of the improved photocatalytic performance of pure g-C_3_N_4_, many strategies have been made to develop cheaper and recyclable catalysts, such as nanostructure design (Sun et al., 2012; Bai et al., 2013; Tahir et al., 2014; Lin et al., 2016; Zhou et al., 2018; Yang et al., 2019), loading with noble metals (Zhang et al., 2016; Duan et al., 2019; Wan et al., 2020) or doping non-metal elements (Guo et al., 2017; Liu et al., 2018; Li et al., 2020), and heterojunction construction (Zhang et al., 2013; Shi et al., 2017; Ji et al., 2018). However, noble metals are rare and too expensive, and their stability is also a big challenge. Doping of non-metal elements would need high temperature and produce many noxious and odorous gases. While nanostructure design usually requires multiple synthetic steps, and templates are also required for special structures. Therefore, the enhanced photocatalytic performance of g-C_3_N_4_–based catalysts is still in urgent demand. As for heterojunction construction*,* the combination of g-C_3_N_4_ with another semiconductor that has well-matched band structures can not only expand the light absorption into the wide absorption region but also be formed between the two components, which effectively guarantee the separation of rapid charges and transfer in the contacted interface. As a visible light–responsive photocatalyst, CdS has also drawn great attention, and successfully applied in the fields of environmental protection, hydrogen evolution, and selective organic synthesis (Liu et al., 2015; Ren et al., 2019; Ai et al., 2020). Coupling g-C_3_N_4_ with CdS could be a feasible route to improve the photocatalytic activity. In previous reports, Chen et al. reported the synthesis of g-C_3_N_4_/CdS composites with a tunable density of CdS nanodots adopting the *in situ* photochemical deposition method (Chen et al., 2019). Cui et al. synthesized C_3_N_4_/CdS composites through a one-step calcination process at high temperatures (Cui, 2015). C_3_N_4_–CdS heterostructures were constructed by a precipitation–deposition route (Fu et al., 2013). All the reported g-C_3_N_4_/CdS composites showed higher photocatalytic activity and stability than individual g-C_3_N_4_ and CdS, ascribing to the synergic effect between g-C_3_N_4_ and CdS, which can effectively promote the charge separation and transfer. Although numerous heterostructure photocatalysts have been designed, the remarkable improvement of the photocatalytic effect has not been obtained. It was probably caused by large bulk volume or unreasonable contacted interfaces among the two or more components in photocatalysts. It has been investigated that the photocatalytic activity could be influenced by the following factors, such as particle sizes, morphology and structures, and preparation methods (Chen et al., 2010). Therefore, in order to optimize the photocatalytic performances of g-C_3_N_4_/CdS hybrids, some important factors should be taken into account, such as effective contact between two components, well-controlled morphology structure and particle size, and sufficiently exposed reactive active sites. However, few works have been found using the chemical deposition of CdS nanoparticles with controllable intensity and particle size onto g-C_3_N_4_ nanosheets to construct heterojunctions.

Herein, we realize the construction of the highly efficient heterostructured photocatalyst for the photocatalytic hydrogen evolution and degradation of pollutant molecules, where CdS nanoparticles with controllable particle sizes are obtained to modify g-C_3_N_4_ nanosheets through a facile chemical deposition process. It is found that the controllable CdS particle size has a significant effect on morphology, specific surface area, light absorption, and photocatalytic activity for prepared hybrids. The hydrogen generation rate of g-C_3_N_4_/CdS hybrids could reach up to 1,070.9 μmol g^−1^ h^−1^. The effects of CdS nanoparticles for the light absorption and photoinduced charge transport, and the enhanced photocatalytic activities were systematically discussed.

## Experimental

### Synthesis

First, g-C_3_N_4_ nanosheets were prepared according to the previous report (Fan et al., 2020). Briefly, the amount of urea was heated at 570°C under air for 3 h, and the yellow product was obtained for further use. Then 10.0 mg of g-C_3_N_4_ nanosheets were dispersed into 20.0 ml aqueous solution containing different amounts of CdCl_2_.2.5H_2_O (with 7.50, 15.0, and 22.5 mg CdCl_2_.2.5H_2_O, respectively) by ultrasonication for 30 min. Then 0.25 ml NH_3_·H_2_O and 5.0 ml thioacetamide (TAA) solution (with 5.5 mg TAA) was added, followed by a water bath at 60°C for 3 h. The resultant products were washed with water and ethanol three times and collected by centrifugation, followed by drying at 60°C for 4 h. After that, the as-prepared products were annealed at 300°C for 2 h in the nitrogen atmosphere by a tubular furnace to improve the crystallinity CdS. The as-prepared g-C_3_N_4_/CdS hybrids were denoted as g-C_3_N_4_/CdS-1, g-C_3_N_4_/CdS-2, and g-C_3_N_4_/CdS-3, respectively.

### Photocatalytic Activity Tests

Photocatalytic degradation of organic pollutes was carried out in a photoreactor system (Xujiang XPA-7) with 5.0 mg of samples dispersed in a 25-ml target molecule solution (2.0 × 10^−5^ M). After that, a 400 W metal halide lamp with a filter (λ ˃400 nm) was irradiated to trigger the photocatalytic reaction. The concentration of the degraded solution was detected by a UV-vis spectrophotometer.

The photocatalytic H_2_ production was measured in a closed quartz reaction system (300 W Xe lamp with a filter as the light source, λ ˃400 nm) using triethanolamine as the sacrificial reagent. 100 ml solution (with 20% triethanolamine and dispersed 10.0 mg samples) was continuously stirred at a fixed speed. The amount of H_2_ was determined by the online gas chromatography (CEAULIGHT, GC-7920). During the photocatalytic tests, a cooling system was used to maintain the temperature constant.

### Characterization

TEM (JEOL, JEM-2100) and HRTEM (FEI, Tecnai G2 F30 S-Twin TEM) were measured for imaging. The phase composition was measured by the Bruker D8 ADVANCE X-ray diffractometer (XRD). BET-specific surface areas and pore structures were determined by a Beishide 3H-2000PS2 system. XPS measurement was performed on a Thermo Scientific ESCALAB 250Xi spectrometer. The photocurrent response experiment was performed on a photoelectrochemical workstation (CIMPS-2, Zahner) with a three-electrode system; 300 W Xe lamp and Na_2_SO_4_ aqueous solution (0.1 M) were used as the light source and electrolyte solution, respectively. The hydroxyl radicals were detected using a fluorescence spectrophotometer (Hitachi F-7000).

## Results and Discussions

### Morphologies and Structural Characterization

**SCHEME 1:**
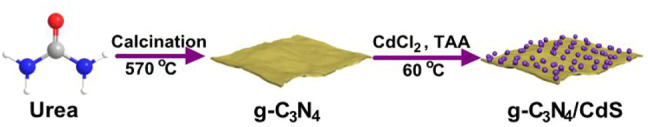
Schematic representation of the synthesis processes of g-C_3_N_4_/CdS hybrids.

**FIGURE 1:**
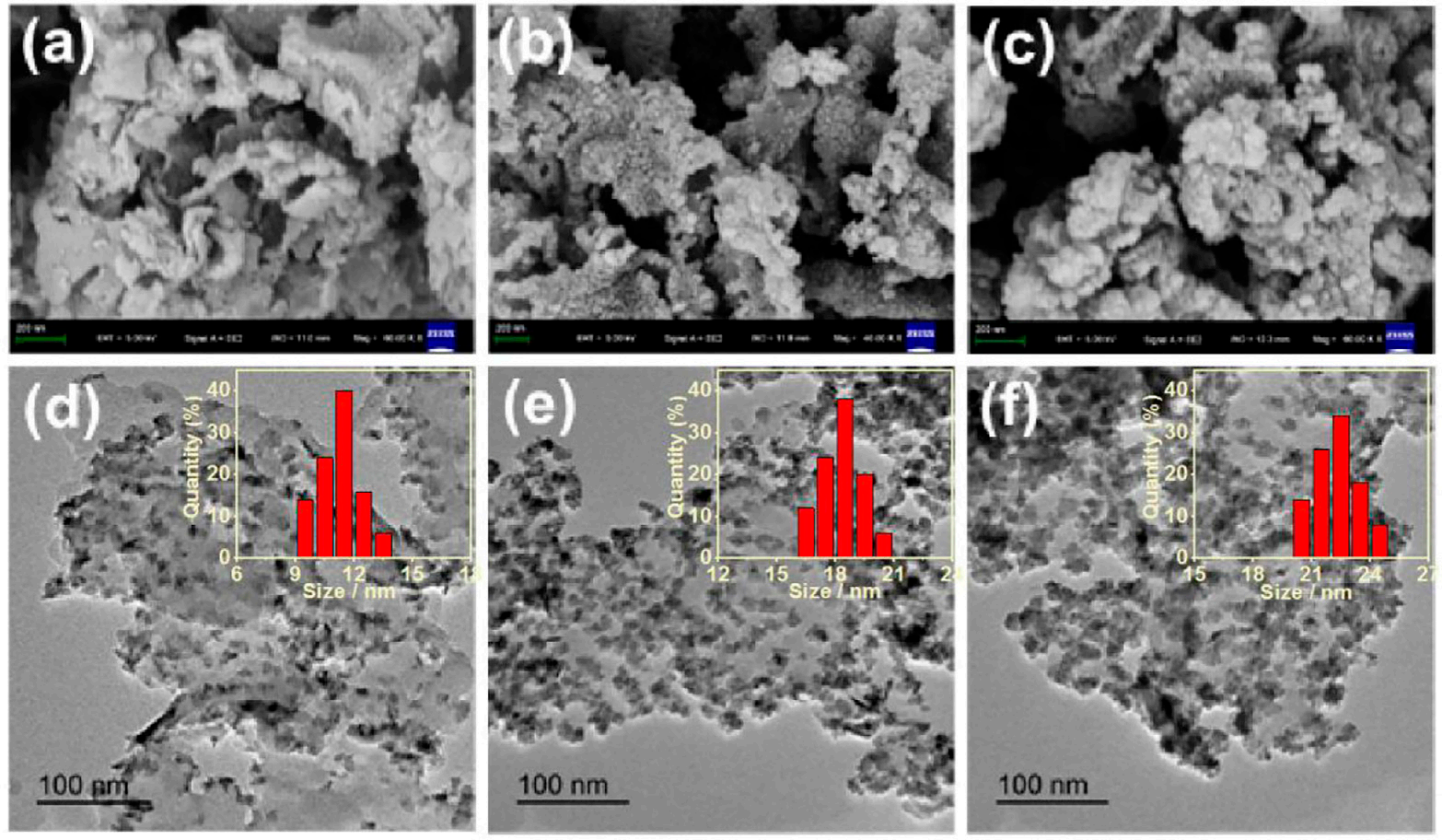
**(A–C)** SEM and **(D–F)** TEM images of (a, d) g-C_3_N_4_/CdS-1 (b, e) g-C_3_N_4_/CdS-2, and (c, f) g-C_3_N_4_/CdS-3 hybrids. Inset: The statistical size distributions of CdS nanoparticles for g-C_3_N_4_/CdS-1/2/3 hybrids.

The synthesis processes of g-C_3_N_4_/CdS hybrids are shown in Scheme 1. The morphology and structure of g-C_3_N_4_/CdS samples were characterized by SEM and TEM, as given in Figure 1. Supplementary Figure S1 is the TEM image of the pure g-C_3_N_4_ sample with a layered structure, which can offer a substrate for the arching of CdS nanoparticles. For the g-C_3_N_4_/CdS samples (Figure 1), it should be noted that the CdS nanoparticles are well loaded onto the g-C_3_N_4_ nanosheets. The amount and particle sizes of CdS nanoparticles can be tuned by changing the amount of the CdCl_2_ precursor. As demonstrated in Figures 1A,D, for the sample g-C_3_N_4_/CdS-1, CdS nanoparticles were evenly dispersed on g-C_3_N_4_ nanosheets with a size narrowed by about 11.5 nm. With increasing the amount of the CdCl_2_ precursor, more nanoparticles with a larger size, up to 18.0 nm, can be observed for sample g-C_3_N_4_/CdS-2 (Figures 1B,E). When the amount of CdCl_2_ was increased to 22.5 mg, for sample g-C_3_N_4_/CdS-3, the size distribution of CdS nanoparticles turns to be wider, and CdS nanoparticles with a diameter of ∼23.5 and ∼8.8 nm can be observed (Figures 1C,F). The statistical size distributions of CdS nanoparticles for g-C_3_N_4_/CdS-1/2/3 hybrids are also given in Figure 1.

**FIGURE 2:**
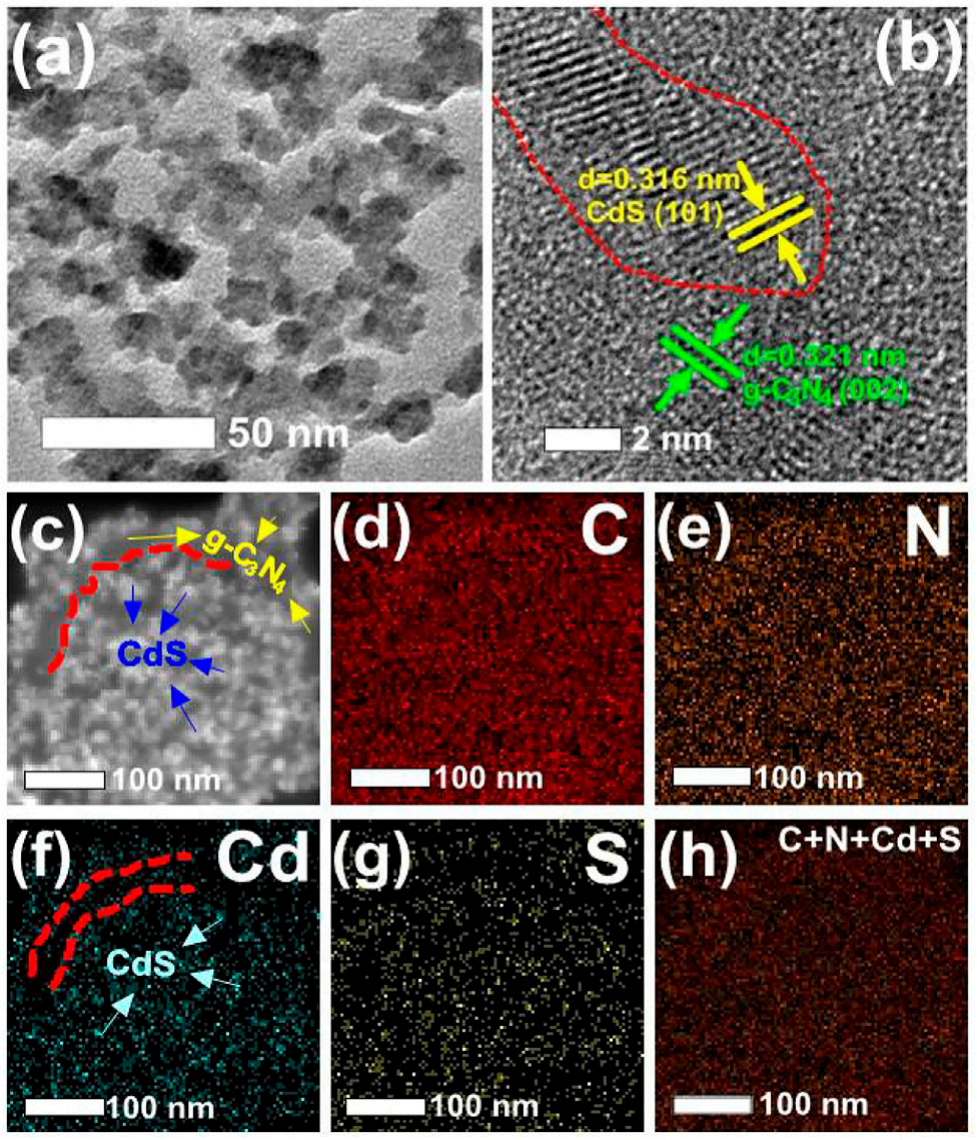
**(A)** TEM, **(B)** HRTEM, and **(C)** HAADF-STEM images of g-C_3_N_4_/CdS-2 hybrids. **(D–G)** Elemental mappings of (d) C, (e) N, (f) Cd, (g) S, and (h) C + N + Cd + S of g-C_3_N_4_/CdS-2 hybrids.

As seen from Figures 2A–C, TEM, HRTEM, and EDX mapping indicated the successful loading of CdS nanoparticles on a g-C_3_N_4_ nanosheet. The TEM image (Figure 2A) shows a high distribution of CdS nanoparticles with average sizes of about ∼18 nm dispersed on g-C_3_N_4_ nanosheets. The HRTEM image (Figure 2B) gives two sets of distinct lattice fringes, whereas the spacing of about 0.316 and 0.321 nm, corresponding to the (101) and (002) planes of CdS and g-C_3_N_4_, respectively (Wang et al., 2017a; Zhang et al., 2017; Chiu et al., 2019). Obviously, EDX mappings demonstrate the existence of C, N, Cd, and S (Figures 2D–H), suggesting the uniform distribution of Cd and S elements from g-C_3_N_4_/CdS (Figure 2C), confirming the good connection between CdS and g-C_3_N_4_.

**FIGURE 3:**
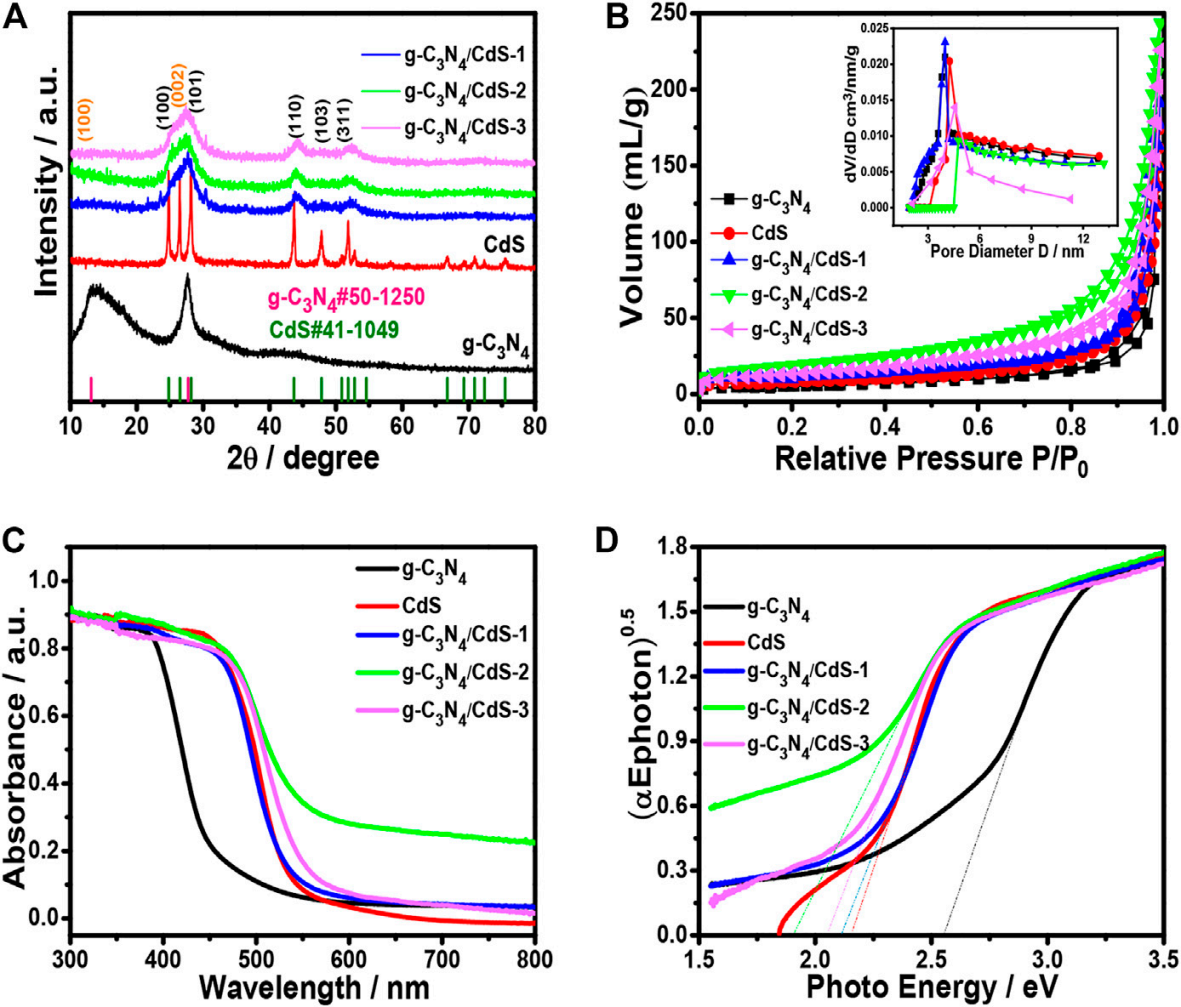
**(A)** XRD patterns and **(B)** nitrogen adsorption–desorption isotherms of g-C_3_N_4_, CdS, and g-C_3_N_4_/CdS. Inset in Figure 3B is the corresponding BJH pore size distribution. **(C)** UV-Vis of absorption spectra and **(D)** the corresponding (αEphoton)^0.5^ vs photon energy curves.

The crystal phases of g-C_3_N_4_, CdS, and g-C_3_N_4_/CdS samples were analyzed by XRD. As shown in Figure 3A, all diffraction peaks for g-C_3_N_4_ and CdS can be observed. The two strong diffraction peaks clearly shown at 12.8^o^ and 27.6^o^ can be ascribed to (100) and (002) crystal planes of g-C_3_N_4_ (PDF#50-1,250), which can be associated with typical in-plane tri-s-triazine and graphitic stacking of g-C_3_N_4_ (Xu et al., 2020). Furthermore, diffraction peaks at 24.8^o^, 26.5^o^, and 28.3^o^ are well matched for those of hexagonal CdS (PDF#41-1,049). The pore structures and specific surface areas for prepared samples were determined by BET measurements. The corresponding results of sample CdS nanoparticles, g-C_3_N_4_ nanosheets, and g-C_3_N_4_/CdS-(1-3) hybrids are given in Figure 3B and Supplementary Figure S2, suggesting the existence of mesoporous pores. The S_BET_ of sample g-C_3_N_4_/CdS-2 is the highest S_BET_ among all the prepared samples, which can provide more reactive sites and ensure better contact catalysts with reagents during the photocatalytic reactions. The light absorption of pristine g-C_3_N_4_, CdS nanoparticles, and the as-prepared g-C_3_N_4_/CdS samples are shown in Figures 3C,D. The absorption around 470 nm is assigned for pristine g-C_3_N_4_. It can be obviously observed that the absorption ranges of prepared g-C_3_N_4_/CdS samples were extended. The resulting values of *E*_g_ for CdS, g-C_3_N_4_, and g-C_3_N_4_/CdS-(1-3) samples were 2.16, 2.60, 2.11, 1.91, and 2.05 eV, respectively. The enhanced absorption for g-C_3_N_4_/CdS is probably due to the formation of heterojunctions. Sample g-C_3_N_4_/CdS-2 with appropriate CdS particle size has the lowest bandgap, the stronger background absorption could be ascribed to the synergistic interaction between g-C_3_N_4_ nanosheets and CdS nanoparticles, and the photoinduced electrons from the LUMO of CdS could adequately inject into the conduction band of g-C_3_N_4_, leading to the reduced initial bandgap values of CdS and g-C_3_N_4_, separately (Al Marzouqi et al., 2019).

**FIGURE 4:**
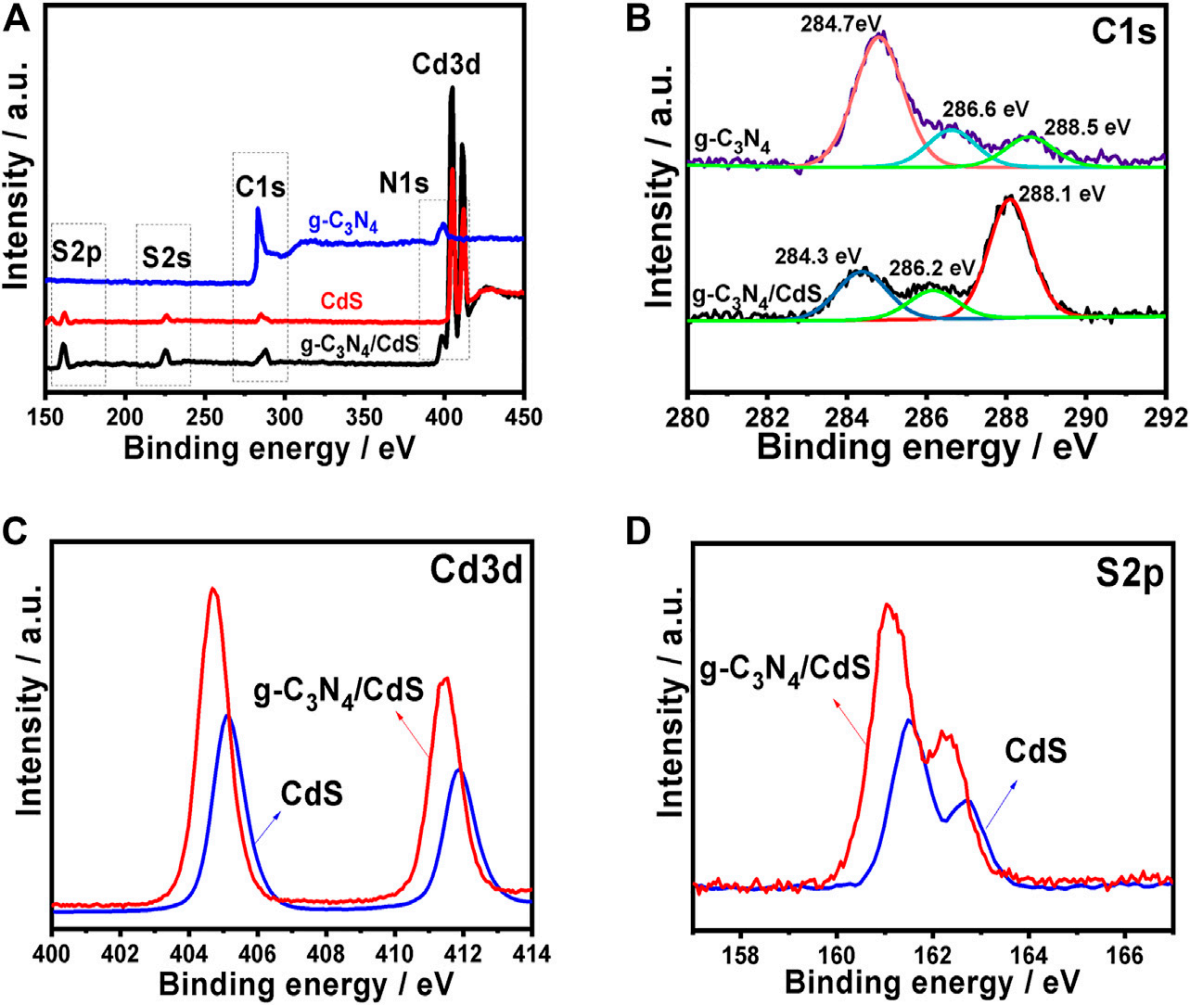
Fully scanned XPS spectra of **(A)** g-C_3_N_4_, CdS, and g-C_3_N_4_/CdS; high-resolution XPS plots of **(B)** C 1 s, **(C)** Cd 3 days, and **(D)** S 2p.

The surface composition of the g-C_3_N_4_, CdS, and g-C_3_N_4_/CdS samples was studied by XPS analyses, as shown in Figure 4. The survey XPS spectra (Figure 4A) provide the C 1s and N 1s peaks for g-C_3_N_4_, as well as S 2p and Cd 3 days peak for CdS. As shown in Figure 4B, the C 1s peak for g-C_3_N_4_/CdS at 284.3 eV, which is assigned to sp^2^ C-C bonds from graphitic carbon. The other two peaks at 286.2 and 288.1 eV are attributed to N-C=N and π-excitation, respectively (Gao et al., 2014; Liang et al., 2019; Xu et al., 2020), These peaks show a slight shift while compared with g-C_3_N_4_. In Figure 4C, the two peaks at 405.5 and 412.2 eV could be ascribed to Cd 3d_5/2_ and Cd 3d_3/2_, of CdS from g-C_3_N_4_/CdS. In addition, the peaks at 161.1 and 162.3 eV are associated with S 2p_3/2_ and S 2p_1/2_ of CdS, respectively (Figure 4D; Ren et al., 2019; Wang et al., 2019; Ai et al., 2020). These peaks show a slight shift while compared with pristine CdS or g-C_3_N_4_, which confirm the interaction between the heterojunctions.

### Photocatalytic Performances and Mechanism Discussion

**FIGURE 5:**
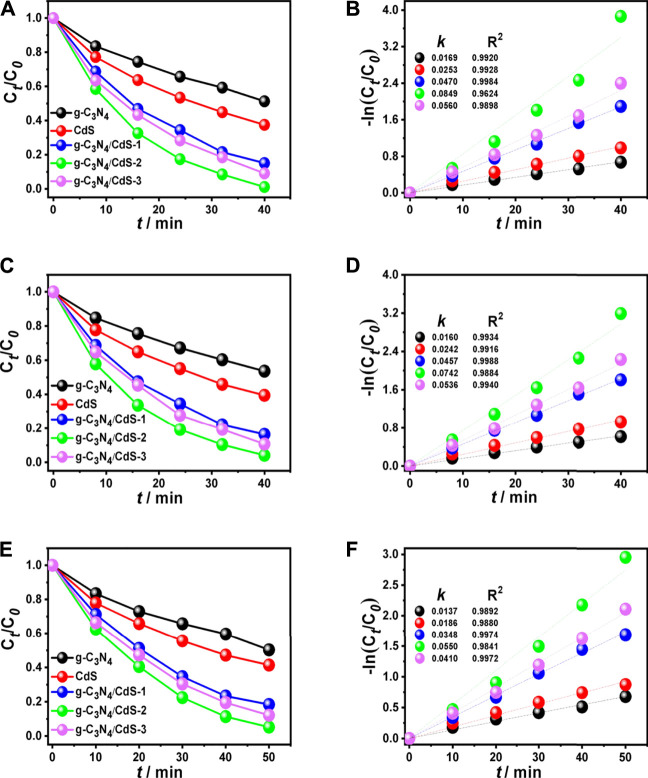
Photocatalytic degradation under visible light irradiation of **(A)** RhB, **(C)** MB, and **(E)** phenol, and **(B,D,F)** corresponding apparent reaction rates using g-C_3_N_4_, CdS, and g-C_3_N_4_/CdS hybrids.

**FIGURE 6:**
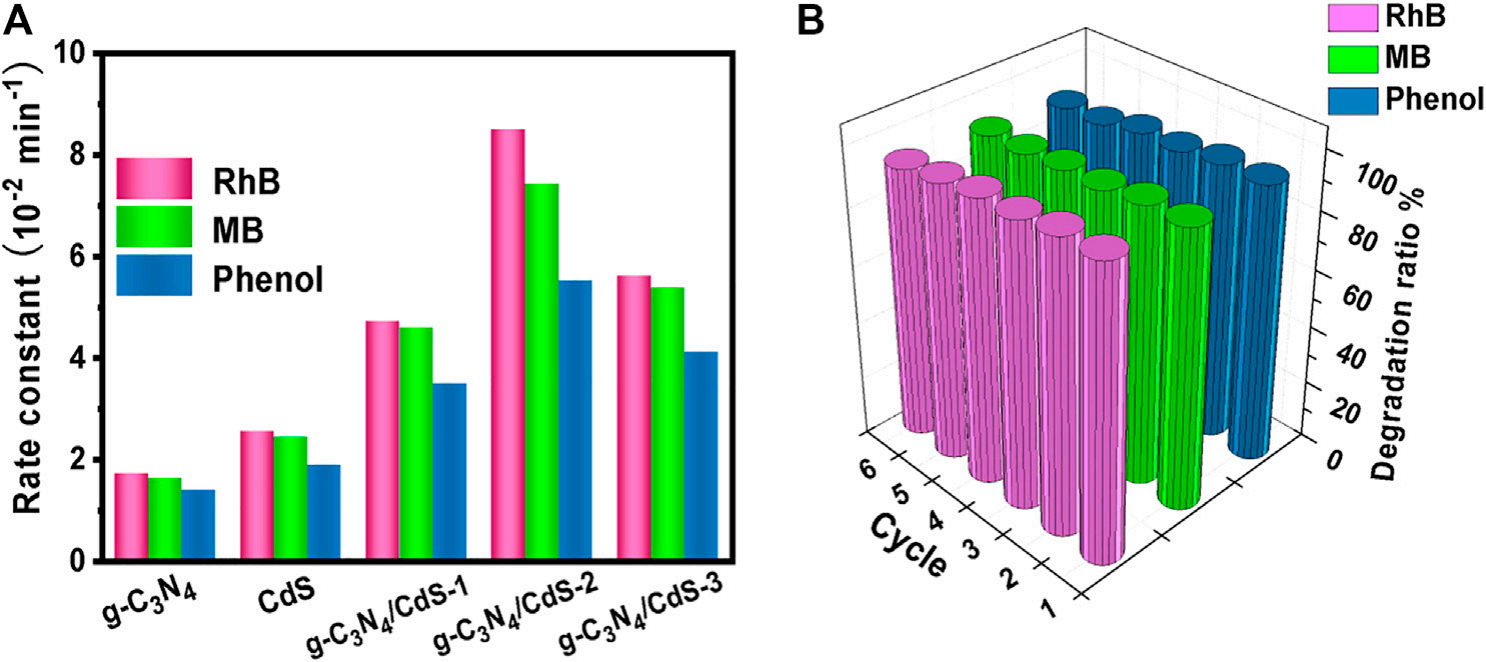
**(A)** Comparison of rate constants of g-C_3_N_4_, CdS, and g-C_3_N_4_/CdS hybrids for organic pollution degradation (RhB, MB, and phenol) under visible light irradiation. **(B)** Recyclability measurements of RhB, MB, and phenol degradation under visible light irradiation by g-C_3_N_4_/CdS hybrids.

The photocatalytic activities of g-C_3_N_4_, CdS, and g-C_3_N_4_/CdS samples were first evaluated with degradation of organic pollution, as given in Figure 5. It can be seen from Figures 5A,B that coupling with CdS shows a significant effect on RhB degradation for g-C_3_N_4_/CdS. The photocatalytic activity shows a trend of initially increasing and then decreasing with the increasing of CdS contents. The degradation efficiency of RhB can reach up to 99% within 40 min for g-C_3_N_4_/CdS-2 hybrids, which is remarkably enhanced than that for individual g-C_3_N_4_ nanosheets and CdS nanoparticles. The photocatalytic rate constant for the prepared g-C_3_N_4_/CdS-2 is 0.0849 min^−1^, about 5.5 times higher than that for g-C_3_N_4_. The photocatalytic results demonstrate that the anchoring of CdS nanoparticles onto g-C_3_N_4_ nanosheets can remarkably be conducive to photocatalytic performance. The enhanced activity for sample g-C_3_N_4_/CdS-2 can be attributed to well-contacted interfaces, effective charge separation, stronger light absorption, and a higher surface area. Furthermore, the TOC analyzer was measured to investigate the mineralization of RhB. The changed TOC values during the photocatalytic process were tested, as shown in Supplementary Table S1 and Supplementary Figure S3. The decayed TOC values suggest that RhB was decomposed into inorganic small molecules. As for MB (Figures 5C,D) and phenol (Figures 5E,F) degradation, g-C_3_N_4_/CdS-2 sample also exhibits much higher activity than others, where 96% of MB and 95% of phenol are degraded within 40 and 50 min, respectively. The degradation rate constants *k* for MB and phenol of prepared samples follow the same order with RhB degradation (Figure 6A). Moreover, the g-C_3_N_4_/CdS-2 sample can be recycled for six cycles without obviously decayed activity, and no scattering of CdS particles was found, indicating their excellent recyclability and stability (Figure 6B and Supplementary Figure S4).

**FIGURE 7:**
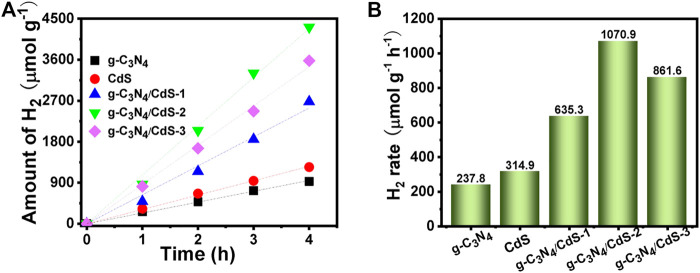
**(A,B)** Photocatalytic hydrogen evolution on g-C_3_N_4_, CdS, and g-C_3_N_4_/CdS hybrids under visible light.

The photocatalytic hydrogen evolution of prepared samples was investigated under visible-light irradiation with triethanolamine as the sacrificial reagent. As shown in Figures 7A,B, within 4 h measurement, pure g-C_3_N_4_ shows poor catalytic activity with the hydrogen generation rate of 237.8 μmol g^−1^ h ^−1^, and the hydrogen generation rate using g-C_3_N_4_ nanosheets is obviously improved after anchoring with CdS. The hydrogen evolution rate is increased first and then decayed with increasing CdS content, and the g-C_3_N_4_/CdS-2 sample exhibits the best hydrogen evolution rate (1,070.9 μmol g^−1^ h ^−1^), about 4 times as high as that for pure g-C_3_N_4_. The photocatalytic performance for g-C_3_N_4_/CdS-2 sample also surpasses the reported g-C_3_N_4_/CdS hybrids (Supplementary Table S2). In addition, the recyclability of photocatalytic hydrogen evolution was also measured, as shown in Supplementary Figure S5a. It can be seen that the hydrogen evolution performance of g-C_3_N_4_/CdS-2 shows no remarkable decline after 12 h, confirming its good stability.

**FIGURE 8:**
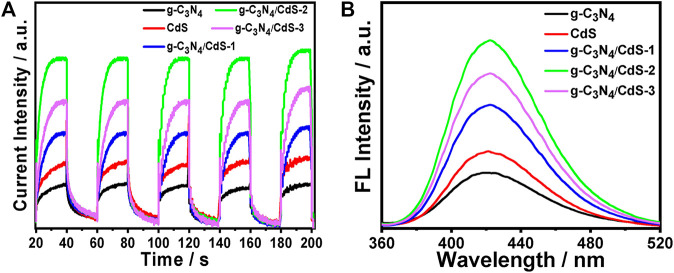
**(A)** Photocurrents of g-C_3_N_4_, CdS, and g-C_3_N_4_/CdS hybrids under visible light irradiation under 0.8 V *versus* Ag/AgCl electrode bias. **(B)** Fluorescence spectra of g-C_3_N_4_, CdS, and g-C_3_N_4_/CdS hybrids in a basic solution of terephthalic acid under visible light irradiation at a fixed time.

**SCHEME 2:**
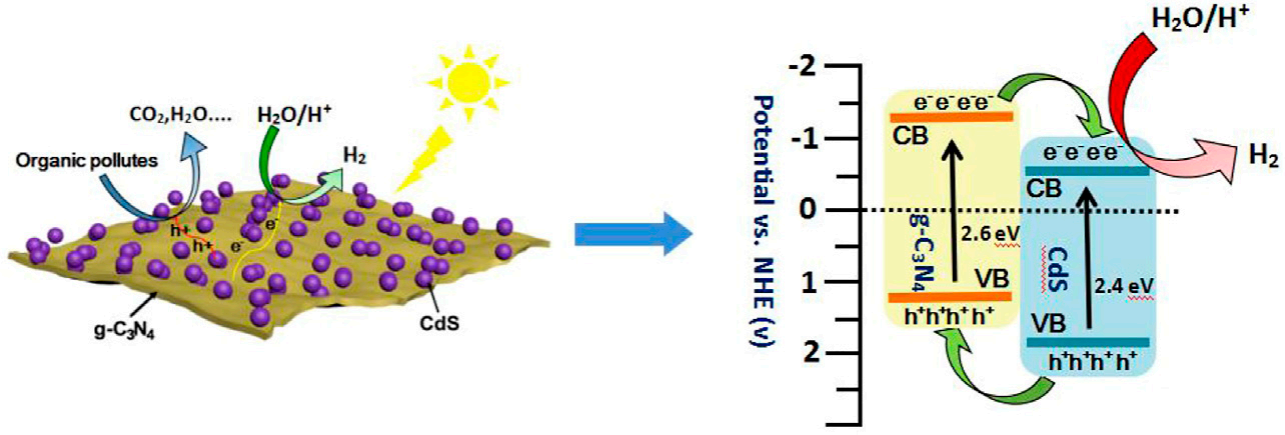
Schematic illustration of band structure diagram and photoinduced carrier transfer of g-C_3_N_4_/CdS hybrids in the piezoelectric field under visible light irradiation.

As observed in Figure 8A, the photocurrent response of prepared samples is detected by photoelectrochemical testing to study the charge separation. It can be found that g-C_3_N_4_/CdS heterostructured samples show much higher photocurrent intensity than individual CdS and g-C_3_N_4_, which can possibly be ascribed to the heterostructure-induced acceleration of charge separation and transfer. (Tian et al., 2017; Ren et al., 2018; Zhu et al., 2019; Xu et al., 2019; Putri et al., 2020). In order to conduct an in-depth study on photocatalytic degradation, the scavenger test was performed using methanol as the scavenger. As shown in Supplementary Figure S5b, the photocatalytic performance of the RhB photodegradation rate is remarkably decreased after adding methanol, suggesting that the photogenerated holes play an important role in the photocatalytic process. In view of the results, a possible mechanism for g-C_3_N_4_/CdS sample with enhanced photocatalytic performance is put forward. The narrowed bandgap semiconductors g-C_3_N_4_ and CdS can be excited under light irradiation. Briefly, the electrons produced from g-C_3_N_4_ will transfer to CdS owing to the well-contacted interface, and the excited holes can move from CdS to the VB of g-C_3_N_4_. Consequently, the separation of photoinduced electrons and holes is effectively improved, and the lifetime of charges is improved (Scheme 2). The transferred strongly oxidizing holes on the VB of g-C_3_N_4_ can not only effectively inhibit photocorrosive damage to CdS during the reaction process but also directly degrade the target molecules. In the process of photocatalytic reaction, the photoexcited electrons may reduce H^+^ into hydrogen. Meanwhile, the accumulated electrons on the surface of CdS can oxidize the adsorbed dissolved O_2_ to produce •O_2_^−^ and subsequently form •OH. The formation of powerful reactive species (•O_2_^−^ and •OH) can effectively degrade the organic pollutant molecules (Huo et al., 2012; Wu et al., 2018). The produced hydroxyl radicals can be measured by the fluorescence method with terephthalic acid as the probe reactant (Xiao et al., 2008; Yu et al., 2009; Wang et al., 2017b). The fluorescence spectra of all samples are shown in Figure 8B, where sample g-C_3_N_4_/CdS-2 can produce the largest amount of hydroxyl radicals. The obtained results are well consistent with those of the photocatalytic tests. The higher photocatalytic activity for g-C_3_N_4_/CdS sample can be ascribed to two main factors: 1) the significantly promoted charge separation by the constructed heterojunctions and 2) the higher specific surface area and excellent dispersion of prepared samples.

## Conclusion

Heterostructured g-C_3_N_4_/CdS hybrids with controllable CdS nanoparticle anchoring on g-C_3_N_4_ nanosheets have been successfully prepared. The as-prepared samples show remarkably improved photocatalytic activity for organic pollute degradation and hydrogen generation under visible light. The experimental results demonstrate that the g-C_3_N_4_/CdS heterostructures exhibited superior photocatalytic hydrogen generation activity than individual g-C_3_N_4_ nanosheets and CdS nanoparticles, and the g-C_3_N_4_/CdS-2 sample has the highest visible light hydrogen generation rate as 1,070.9 μmol h^−1^ g^−1^, which is 4 times higher than that for pure g-C_3_N_4_. The enhanced activity and stability are attributed to the intimated heterojunction between g-C_3_N_4_ nanosheets and CdS nanoparticles, which promotes interfacial charge separation and transportation. Finally, the well-dispersed CdS nanoparticles offer more reactive sites. The g-C_3_N_4_/CdS hybrids demonstrate high photocatalytic activity, stability, and recyclability, and hold great promise for practical application. This work could provide new perspective into the construction and manufacture of heterojunctions with highly efficient charge separation and migration for the solar energy conversion.

## Data Availability

The original contributions presented in the study are included in the article/Supplementary Material; further inquiries can be directed to the corresponding authors.
