# Supplementary material for: Fabrication of g-C3N4 Nanosheets Anchored With Controllable CdS Nanoparticles for Enhanced Visible-Light Photocatalytic Performance
Source: Front Chem. 2021 Oct 14;9:746031. doi: 10.3389/fchem.2021.746031 (PMC8553295; doi:10.3389/fchem.2021.746031)
Supplement: Supplementary file 1 [file Table1.DOCX]

Supplementary Material

**Fabrication of g-C_3_N_4_ nanosheets anchored with controllable CdS nanoparticles for enhanced visible-light photocatalytic performance**

**Minggui Wang^1,2^, Min Wang^1^, Fang Peng^1^, Xiaohuan Sun^2^, Jie Han ^2*^**

^1^ Guangling College, Yangzhou University, Yangzhou, Jiangsu, 225002, P. R. China

^2^ School of Chemistry and Chemical Engineering, Yangzhou University, Yangzhou, Jiangsu, 225002, P. R. China

*E-mail*: hanjie@yzu.edu.cn;


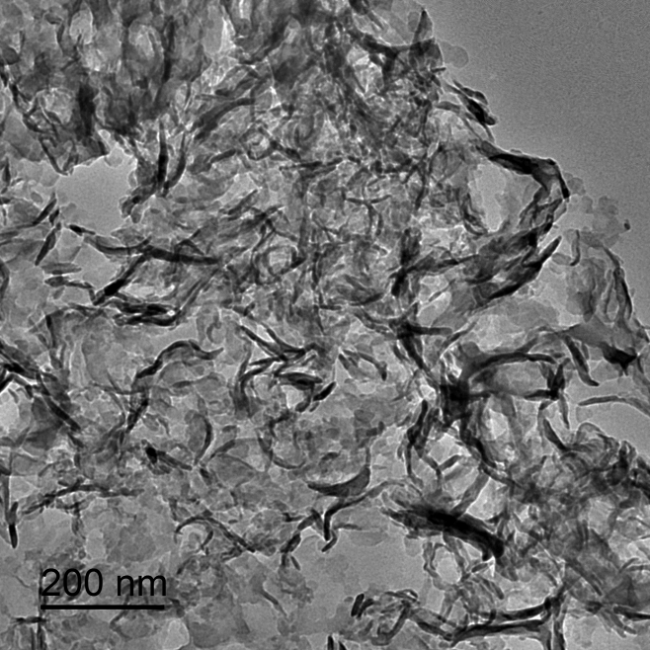


***Fig. S1.*** TEM image of g-C_3_N_4_ nanosheets.

***Fig. S2.*** Specific surface area and pore volume values of as-prepared samples.

***Fig. S3.*** Evolution of RhB concentration and TOC values with visible light irradiation time by g-C_3_N_4_/CdS-2 hybrids.


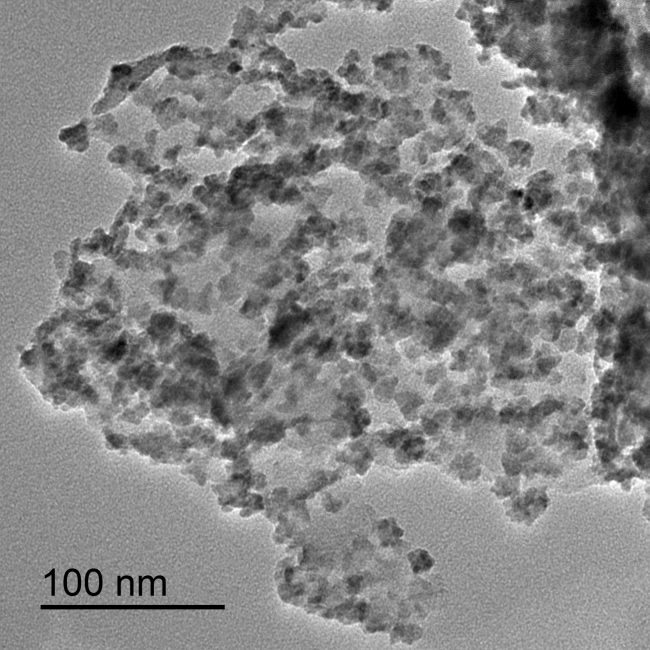


***Fig. S4.*** TEM image of g-C_3_N_4_/CdS-2 hybrids after recycled for six cycles.

***Fig. S5***. (a) Stability test of photocatalytic H_2_ evolution of g-C_3_N_4_/CdS-2. (b) Comparison of photocatalytic RhB degradation over g-C_3_N_4_/CdS hybrids under visible light irradiation with or without hole scavenger.

**Table S1** TOC result for the RhB degradation using g-C_3_N_4_/CdS-2 as catalyst under visible light.

| **Time** (min) | 0 | 20 | 40 | 60 | 100 |
| --- | --- | --- | --- | --- | --- |
| **TOC** (ppm) | 12.10 | 8.244 | 7.102 | 6.352 | 3.864 |

**Table S2** Comparison of the photocatalytic activity of different g-C_3_N_4_/CdS photocatalysts.

| **Sample** | ***k* (min^-1^)** | **HER**  **(μmol h^-1^ g^-1^)** | **irradiation** | **organic dye** | **ref** |
| --- | --- | --- | --- | --- | --- |
| g-C_3_N_4_/BiOBr/CdS composite | 0.0317 | ~ | visible light | tetracycline | 1 |
| CdS/g-C_3_N_4_ nanocomposites | 0.0086 | ~ | visible light | RhB | 2 |
| g-C_3_N_4_/CdS hollow nanotube | ~ | 392.8 | visible light | ~ | 3 |
| CdS/g-C_3_N_4_ heterojunction | ~ | 97.0 | visible light | ~ | 4 |
| g-C_3_N_4_/CdS hybrids | 0.0849 | 1070.9 | visible light | RhB | this work |

**References**

1. Perumal, K., Shanavas, S., Karthigeyan, A., Ahamad, T., Alshehri, S. M., and Murugakoothan, P. Hydrothermal assisted precipitation synthesis of highly stable g-C_3_N_4_/BiOBr/CdS photocatalyst with enhanced visible light photocatalytic degradation of tetracycline. *Diam. Relat. Mater.* **2020**, 110, 108091.
2. Wang, D. S., Xu, Z. X., Luo, Q. Z., Li, X. Y., An, J., Yin, R., and Bao, C. Preparation and visible-light photocatalytic performances of g-C_3_N_4_ surface hybridized with a small amount of CdS Nanoparticles. *J. Mater. Sci*. **2016**, 51, 893-902.
3. Li, G. Q., Liang, H. O., Xu, G. R., Li, C. P., and Bai, J. Controllable synthesized heterojunction hollow nanotube of g-C_3_N_4_/CdS: Enhance visible light catalytic performance for hydrogen production. *J. Phys. Chem. Solids* **2020**, 145, 109549.
4. Güy, N. Directional transfer of photocarriers on CdS/g-C_3_N_4_ heterojunction modified with Pd as a cocatalyst for synergistically enhanced photocatalytic hydrogen production. *Appl. Surf. Sci*. **2020**, 522,146442
